# Sevoflurane-Induced Down-regulation of Hippocampal Oxytocin and Arginine Vasopressin Impairs Juvenile Social Behavioral Abilities

**DOI:** 10.1007/s12031-014-0468-3

**Published:** 2014-11-25

**Authors:** Zhi-Bin Zhou, Xiao-Yu Yang, Bao-Long Yuan, Li-Jun Niu, Xue Zhou, Wen-Qi Huang, Xia Feng, Li-Hua Zhou

**Affiliations:** 1 Department of Anesthesiology, The First Affiliated Hospital of Sun Yat-Sen University, No. 58 Zhongshan 2nd Road, Guangzhou, China; 2 Department of Anatomy, Zhong Shan Medical College, Sun Yat-sen University, No. 74 Zhongshan 2nd Road, Guangzhou, China

**Keywords:** Sevoflurane, Oxytocin, Arginine vasopressin, Hippocampus, Social behavior

## Abstract

Cumulative evidence indicates that early childhood anesthesia can alter a child’s future behavioral performance. Animal researchers have found that sevoflurane, the most commonly used anesthetic for children, can produce damage in the neonatal brains of rodents. To further investigate this phenomenon, we focused on the influence of sevoflurane anesthesia on the development of juvenile social behavioral abilities and the pro-social proteins oxytocin (OT) and arginine vasopressin (AVP) in the neonatal hippocampus. A single 6-h sevoflurane exposure for postnatal day 5 mice resulted in decreased OT and AVP messenger RNA (mRNA) and protein levels in the hippocampus. OT and AVP proteins became sparsely distributed in the dorsal hippocampus after the exposure to sevoflurane. Compared with the air-treated group, mice in the sevoflurane-treated group showed signs of impairment in social recognition memory formation and social discrimination ability. Sevoflurane anesthesia reduces OT and AVP activities in the neonatal hippocampus and impairs social recognition memory formation and social discrimination ability in juvenile mice.

## Introduction

According to retrospective clinical data, children who receive general anesthesia under the age of 2 have twice the incidence of developing autistic symptoms that cause learning disabilities, such as hyperactivity and attention deficit, at a later age (Sprung et al. 2012; Wilder et al. 2009). In animal studies, extensive neural apoptosis was found in neonatal mouse brains after 6 h of 3 % sevoflurane anesthesia. Compromised responses to stress conditions and abnormal social behavioral changes resembling autism were subsequently observed when these mice reached adulthood (Satomoto et al. 2009). Previous research showed that neonatal exposure to 2.3 % sevoflurane for 6 h resulted in the retardation of the sensorimotor reflex development of rats, but the spatial navigation ability of the rats was intact (Feng et al. 2012). Given that sevoflurane exerts multifactorial neurobehavioral impacts, social memory development could possibly be affected as well.

In a genomic microarray analysis conducted by Pan et al. (2011), among the 28,000 *Rattus* genes investigated, oxytocin (OT) and arginine vasopressin (AVP) were found to be significantly down-regulated after sevoflurane exposure. Known as neurohypophysial hormones, OT and AVP are also important neurotransmitters that are closely related to social memory development. Ample studies have confirmed that oxytocinergic and vasopressinergic fibers and their associated receptors exist in the mammalian hippocampus (Feldman et al. 1997; Knobloch et al. 2012; Stoop 2012) and build up the learning and memory functions in social behavioral formation (Bielsky et al. 2004; Stoop 2012). Therefore, we speculate that sevoflurane may affect social memory development in juvenile mammals by regulating OT and AVP expression in the hippocampus.

To investigate this hypothesis, we exposed neonatal mice to sevoflurane and assessed the transcriptional and translational changes of OT and AVP after anesthesia. Social recognition and discrimination abilities were examined to determine whether a single early exposure of sevoflurane could have long-term deleterious effects on juvenile social behavioral development.

## Methods

### Animals

This study was approved by the Institutional Animal Care and Use Committee at Sun Yat-Sen University (Guangzhou, Guangdong, China). Animals were obtained from the Experimental Animal Center of Sun Yat-Sen University. To minimize the number of animals used and their suffering, 48 C57BL male mice (from eight litters) were used. They were kept in a 12-h light–dark cycle environment at 25 °C with access to water and food ad libitum.

### Sevoflurane Exposure

Mice at postnatal day 5 (PN5d; 3–5 g) were randomly allocated into the air-treated (control; *n* = 24) and sevoflurane-treated (sevo; *n* = 24) groups. Mice in the sevo group were placed in a transparent plastic container filled with 2.3 % sevoflurane. Sevoflurane was continuously supplied by a vaporizer with a fresh airflow of 2 L min^−1^. The concentrations of sevoflurane, oxygen and carbon dioxide were monitored by a gas analyzer (Detex-Ohmeda, Louisville, KY, USA) in real time. The temperature in the box was controlled at 38 °C using an infrared heater (NPS-A3 heater, Midea Co., Guangdong, China). After 6 h of sevoflurane exposure, mice were again exposed to air and then placed back into their maternal cages. Mice of control group were exposed to air in the same apparatus for 6 h.

### Social Behavioral Tests

At postnatal day 20, the mice were already capable of social interaction but had not yet reached puberty (the sexual maturation time for C57BL mice is estimated to be 5–8 weeks postnatally) (Pinter et al. 2007). Behavioral tests were employed to examine their social recognition and discrimination abilities (Winslow 2003). All mice used in this experiment were aged postnatal day 20 (including the female stimulus mice), and six mice were allocated into each group.

### Recognition Trial

**Fig. 1:**
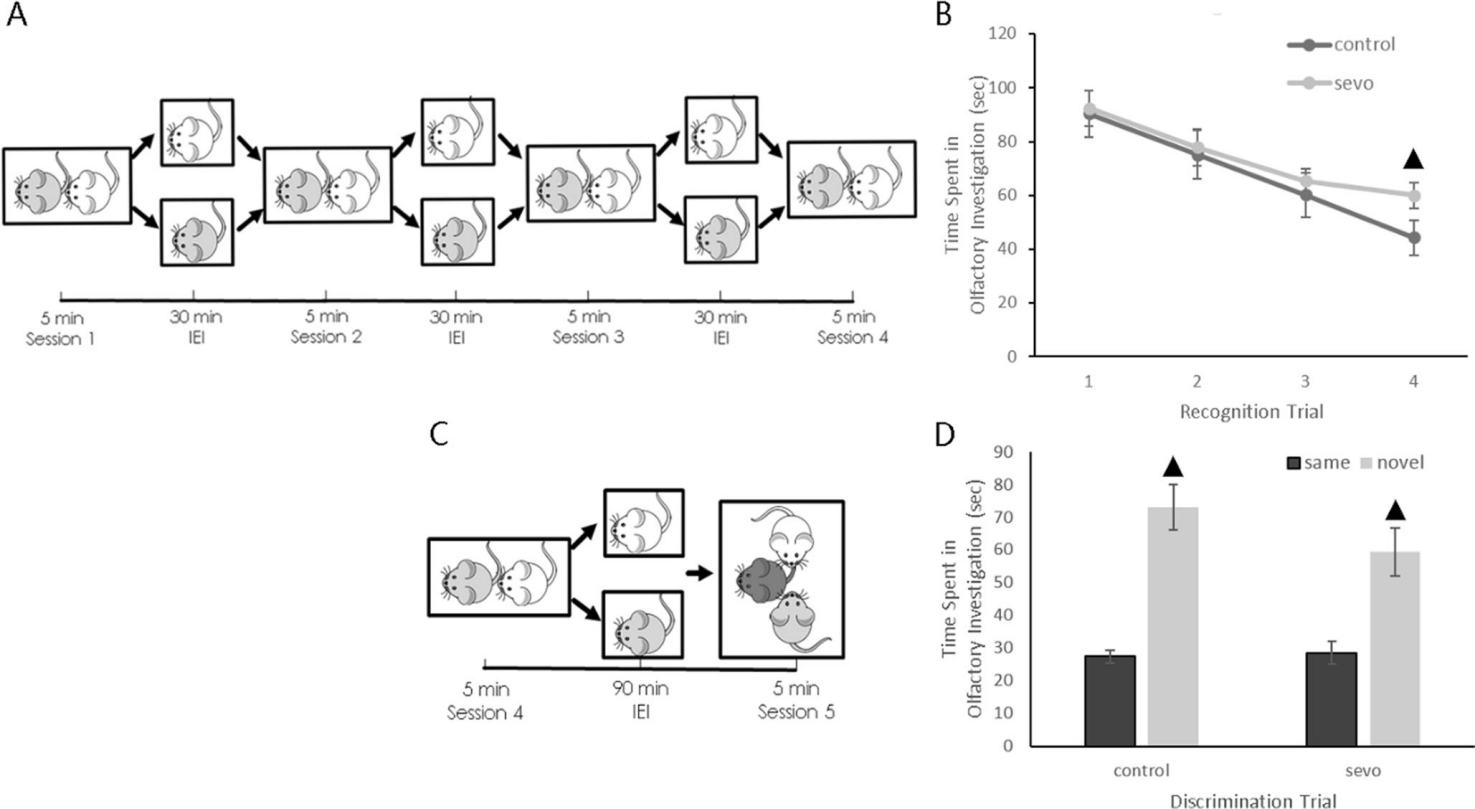
Illustration of the social recognition and social discrimination trials and raw results data. **a** Illustration of the social recognition paradigm. **b** Raw data of the olfactory investigation times in each session of the social recognition trial (^▲^ *P* < 0.001 compared between groups). **c** Illustration of the social discrimination paradigm. **d** Raw data of the olfactory investigation times in session 5 of the social discrimination trial (^▲^ *P* < 0.001 compared between the time spent investigating the same stimulus mouse and that of the novel stimulus mouse). All results are presented as the mean ± SD

The test cage (20 × 20 × 20 cm) was located in a fixed position in a quiet, moderately lighted room. Before the trial, the subject mouse was placed into the test cage for a habituation period of 30 min. Then, a stimulus mouse was introduced into the test cage for a 5-min familiarization period (session 1). The stimulus mouse was positioned along the wall furthest from the test subject. After 5 min, the stimulus mouse was removed from the test cage. The mice were separated for a 30-min inter-encounter interval (IEI), and then the same stimulus mouse was again introduced into the test cage for another 5 min familiarization period (session 2). The prior process was repeated until each subject mouse had encountered its corresponding stimulus mouse four times (as depicted in Fig. 1).

During each familiarization period, the amount of time the subject mouse spent investigating the stimulus mouse was recorded. The investigation time was counted as any time when the subject mouse used its nose to inspect any portion of body of the stimulus mouse within 2 cm.

### Discrimination Trial

The last familiarization (session 4) was followed by an extended IEI of 90 min. After this extended IEI, the same stimulus mouse and a novel stimulus mouse were introduced simultaneously into the test cage for a 5-min discrimination period.

During the discrimination period, the amount of time the subject spent investigating the same stimulus mouse and the novel stimulus mouse were recorded, respectively. The investigation criteria were the same as in recognition trial.

Raw data from both social recognition and social discrimination trials were presented as the Ratio of Investigation Duration (RID) in Table 2. RIDr (recognition) represents the establishment of social memory formation. RIDr equals the amount of time the subject spent investigating the same stimulus mouse in the present session divided by that of the previous session. The lower the RIDr value, the more developed the subject’s social memory formation. RIDd (discrimination) represents the social discrimination ability. RIDd equals the amount of time the subject spent investigating the same stimulus mouse divided by that spent investigating the novel stimulus mouse in the discrimination period. The lower the RIDd value, the stronger the subject’s social discrimination ability.

### Real-Time Quantitative PCR for OT and AVP mRNA Levels

Four hours after sevoflurane exposure, mice of the control (*n* = 6), and sevo (*n* = 6) groups were deeply anesthetized with 10 % chloral hydrate (360 mg/kg, i.p.). Bilateral hippocampal tissue was dissected out on ice and then transferred to EP tubes that were precooled with liquid nitrogen. Total RNA was extracted using RNAiso Plus (Takara, Japan) and stored at −80 °C for later use. The concentration and purity of RNA were measured under a microvolume UV spectrophotometer (NanoDrop 2000, Thermo Fisher Scientific, USA). According to the measured concentrations, all samples were diluted with RNase-free water to 1 μg/μl for reverse transcription. According to the instructions of the M-MLV reverse transcriptase reaction kit (Invitrogen, USA), a 20-μl reaction volume was prepared for 1 μg total RNA. According to the instructions of the SYBR Green quantitative polymerase chain reaction (qPCR reaction kit; Invitrogen, USA), a 25-μl reaction volume was prepared for qPCR. The primer sequences used for qPCR are listed in Table 1.

**Table 1:** Calculated RID results in the social recognition and discrimination trials of juvenile mice

	IEI (min)	RIDr		RIDd	
		Control (*n* = 6)	Sevo (*n* = 6)	Control (*n* = 6)	Sevo (*n* = 6)
Social recognition paradigm	30 (session 1)	–	–	–	–
	30 (session 2)	0.83 ± 0.06	0.84 ± 0.03	–	–
	30 (session 3)	0.80 ± 0.08	0.84 ± 0.05	–	–
	30 (session 4)	0.74 ± 0.06	0.91 ± 0.05^▲^	–	–
Social discrimination paradigm	90 (session 5)	–	–	0.37 ± 0.04	0.49 ± 0.07^▲^

All results are presented as the mean ± SD

*RIDr* recognition ratio of investigation duration, *RIDd* discrimination ratio of investigation duration

^▲^ *P* < 0.001 compared between groups)

### Western Blot Analysis for OT and AVP Protein Levels

Four hours after sevoflurane exposure, bilateral hippocampal tissue was dissected out as described above (control group, *n* = 6; sevo group, *n* = 6) and immediately homogenized with 100 mg/ml RIPA Lysis Buffer (Shenergy Biocolor Co., China) and 1 % (*v*/*v*) PMSF (Shenergy Biocolor Co., China). After 20 min of centrifugation at 13,000×*g* at 4 °C, the supernatant was aspirated and stored at −80 °C for further use. The extracted hippocampal proteins were electrophoresed using a 5 % stacking gel at 80 V for 20 min and 12 % separating gel at 120 V for 40 min and were then transferred onto polyvinylidene fluoride membranes (Pall Co., USA). The blots were immunoreacted with anti-OT (1:500, AB2078, Abcam, UK), anti-AVP (1:500, AB48322, Abcam, UK), and anti-GAPDH (1:1000, SC25778, Santa Cruz, USA) antibodies. The ECL-PLUS Reagents Kit (Com Win Biotech Co., Ltd, China) was utilized to detect the bands. The results were examined under a digital imaging system (ImageQuant Las4000 mini, General Electric, USA). The optical density (OD) was then analyzed with Image J software.

### Immunohistochemical Analysis for OT and AVP Distribution

Four hours after sevoflurane exposure, mice of the control (*n* = 6) and sevo (*n* = 6) groups were executed as described above and transcardially perfused with normal saline until the liver and lung appeared translucent. The mice were continuously perfused with 4 % paraformaldehyde in 0.1 M PB (NaH_2_PO_4_·2H_2_O, 2.96 g; Na2HPO_4_·12H_2_O, 29 g dissolved in 1 l water) for 10–20 min. The brains were then removed for paraformaldehyde fixation overnight. After dehydration and paraffin embedding, coronal hippocampal sections (5 μm thick) were sliced, deparaffinized, and hydrated. Antigen retrieval was performed with EDTA solution (Beyotime, China). Sections were incubated with anti-OT (1:100; AB2078, Abcam, UK) and anti-AVP (1:100; AB68669, Abcam, UK) antibodies. The final results were examined under a microscope imaging system (DM 2500B, Leica, Germany). Image-Pro Plus Software was utilized to count the number of positive particles in the area of dorsal hippocampal region.

### Statistical Analysis

GAPDH was used as the internal control. The Livak Method (2^−△△Ct^ method) was employed for qPCR data analysis. Briefly, an *A* value was calculated in each sample (*A* = Ct of the target gene −Ct of GAPDH), then 2^−*n*^ was calculated to represent the fold change of gene expression in the sevo group compared with the control group (*n* = *A* value of sevo group − *A* value of control group). For western blot analysis, the ratio of the optical densities (OD of the target protein divided by that of GAPDH) was calculated to represent the target protein level. All data were described as the mean ± standard deviation. IBM SPSS Statistics 20 software was used for statistical analysis. Comparisons of the mean values between groups were performed using independent sample *t* tests. *P* values of ≤0.05 were considered statistically significant.

## Results

### Social Behavioral Tests

The results of the social recognition trial indicated that after several familiarization periods, social recognition memory was formed in both control and sevo mice, as could be seen from the decline in the olfactory investigation time. However, compared with the air-treated mice in session 4, mice in the sevoflurane-treated group still maintained a relatively high investigative interest, with a high RIDr value (Fig. 1; Table 2), suggesting that the formation of social memory in the sevoflurane-treated mice is not as well established as that in the controls.

**Table 2:** Forward and reverse primers used for quantitative PCR

Gene symbol	Orientation	Primer sequence (5′→3′)
OT	Forward	CCTACAGCGGATCTCAGACTGA
	Reverse	TCAGAGCCAGTAAGCCAAGCA
AVP	Forward	TCGCCAGGATGCTCAACAC
	Reverse	TCCGAAGCAGCGTCTTGG
GAPDH	Forward	CCATCACCATCTTCCAGGAGCGAG
	Reverse	GATGGCATGGACTGTGGTCATGAG

*OT* oxytocin, *AVP* arginine vasopressin, *GAPDH* glyceraldehyde-3-phosphate dehydrogenase

In the subsequent social discrimination trial, sevoflurane-treated mice had a higher RIDd value, suggesting a compromised social discrimination ability after neonatal sevoflurane exposure.

### Real-Time Quantitative PCR for OT and AVP mRNA

**Fig. 2:**
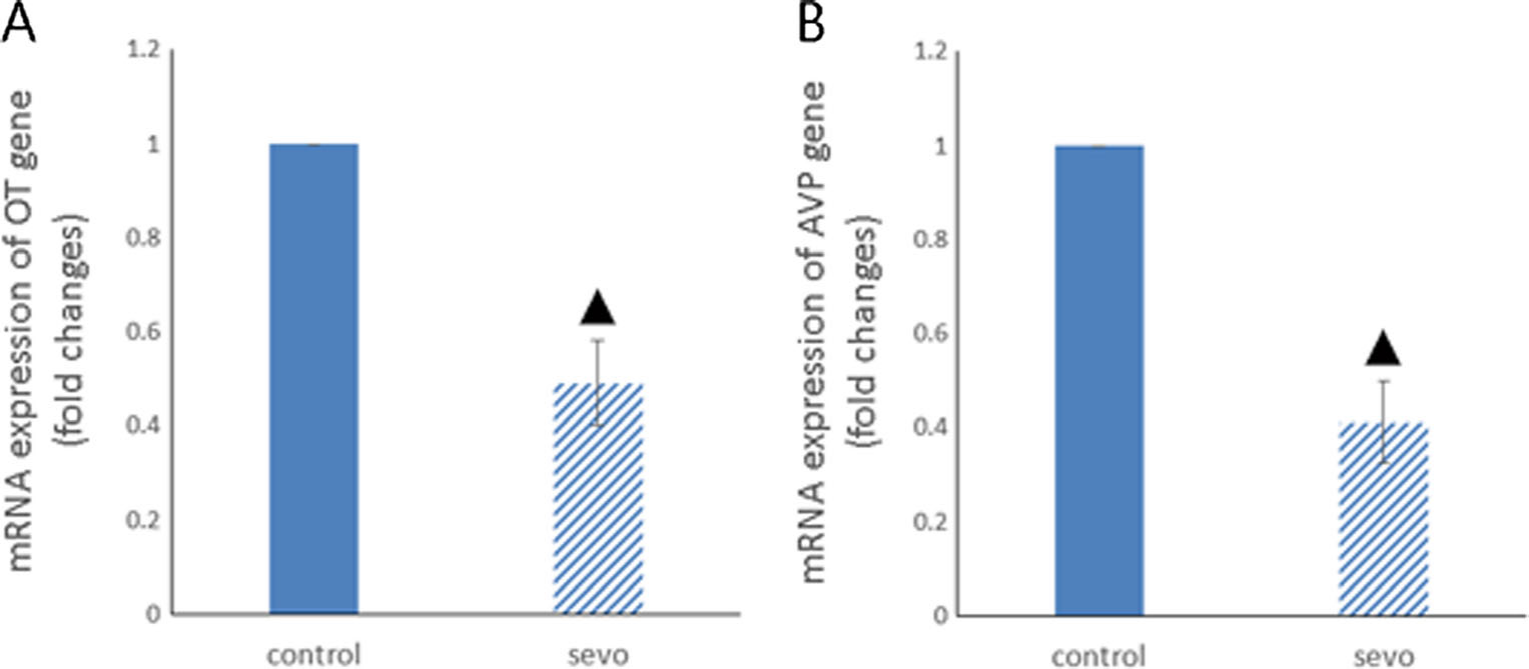
qPCR results for OT and AVP. **a** Hippocampal OT gene transcription levels (^▲^ *P* < 0.001 compared between groups). **b** Hippocampal AVP gene transcription levels (^▲^ *P* < 0.001 compared between groups)

The results of qPCR showed the down-regulation of OT and AVP messenger RNA (mRNA) levels in the mouse hippocampus 4 h after sevoflurane exposure (Fig. 2).

### Western Blot Analysis for OT and AVP Protein Levels

**Fig. 3:**
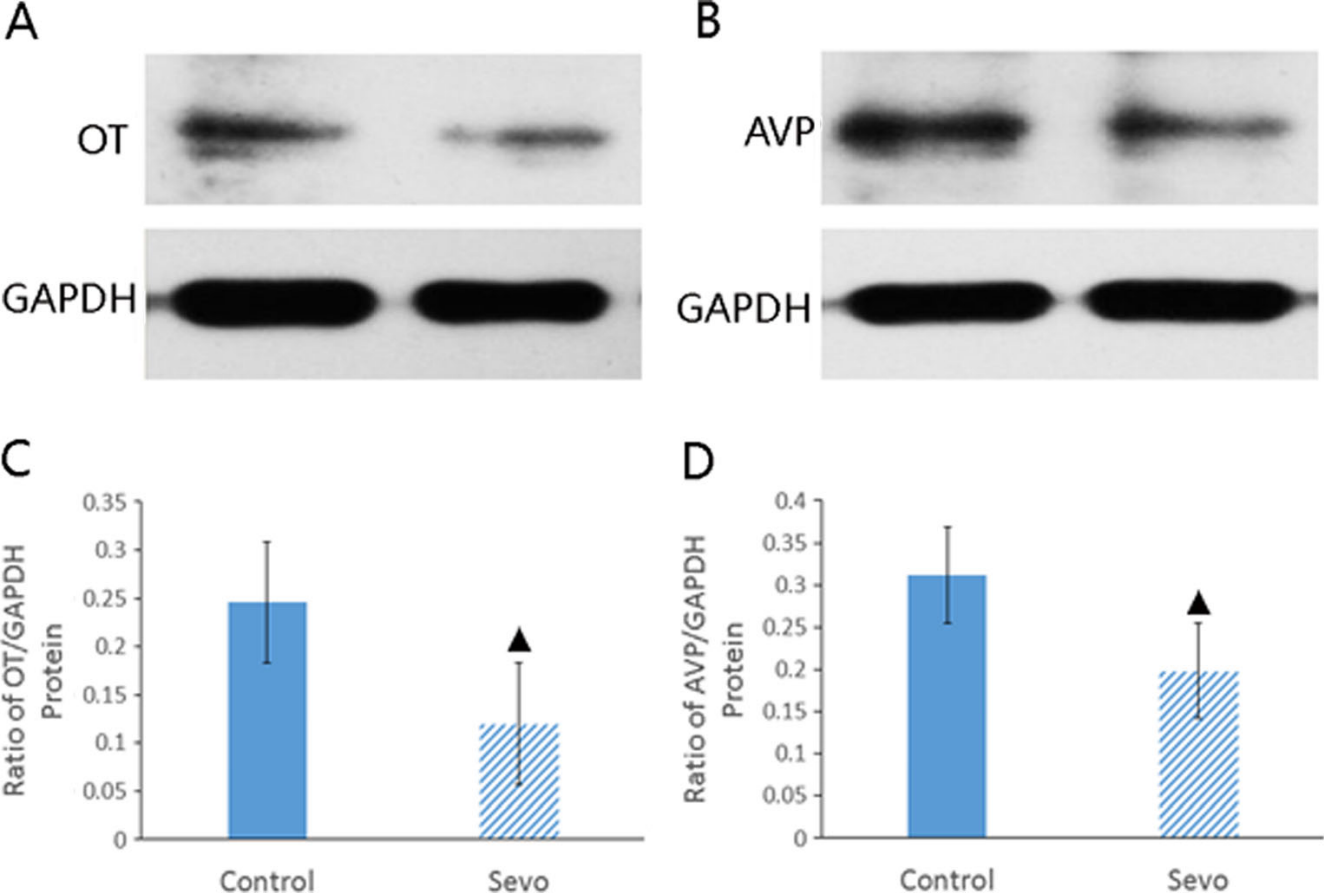
Western blot results for OT and AVP. **a** Western blot results for OT. **b** Western blot results for AVP. **c** Hippocampal OT protein levels (^▲^ *P* < 0.01 compared between groups). **d** Hippocampal AVP protein levels (^▲^ *P* < 0.01 compared between groups)

Four hours after sevoflurane exposure, OT and AVP protein levels were decreased in the mouse hippocampus (Fig. 3).

### Immunohistochemical Analysis for OT and AVP Distribution

**Fig. 4:**
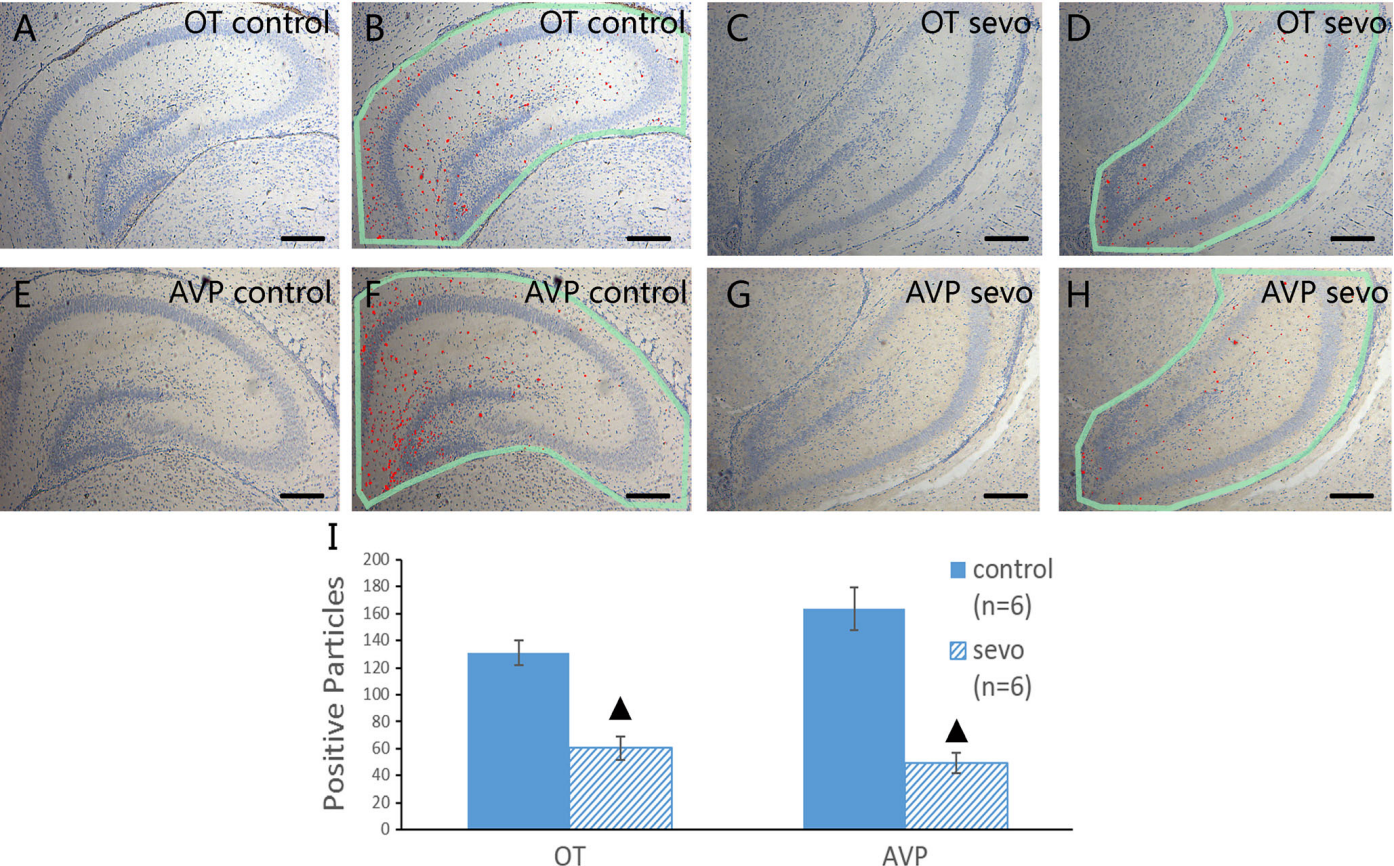
Immunohistochemical staining of OT and AVP in the dorsal hippocampus. **a**, **b** OT immunohistochemical staining results and the corresponding marking illustrations of positive particles in the air-treated group. **c**, **d** OT immunohistochemical staining results and the corresponding marking illustrations of positive particles in the sevoflurane-treated group. **e**, **f** AVP immunohistochemical staining results and the corresponding marking illustrations of positive particles in the air-treated group; **g**, **h** AVP immunohistochemical staining results and the corresponding marking illustrations of positive particles in the sevoflurane-treated group. (×100; *scale bar*, 200 μm, ^▲^ *P* < 0.001 compared between groups)

The results of immunohistochemical staining showed that OT- and AVP-positive particles were mainly located near the dentate gyrus and the inner part of the CA1 region in the dorsal hippocampus. Four hours after sevoflurane exposure, both OT- and AVP-positive particles decreased and became sparsely distributed (Fig. 4).

## Discussion

In previous studies, after sevoflurane exposure, abnormal morphological changes of hippocampal neurons were observed, accompanied by an upregulation of the cell apoptosis marker activated caspase-3 protein. Activated caspase-3 protein reached its peak level at 4–6 h after the exposure (Feng et al. 2012). As a result, we assumed that 4 h after exposure was the time when most hippocampal neurons had undergone vast damage and were at their most vulnerable state. Thus, changes in the OT and AVP levels, if any, could likely be observed at this time point.

One study showed that intracerebral administration of oxytocin receptor agonists reduced the activation of caspase-3 caused by sevoflurane, which indicated a potential neuroprotective role of oxytocin (Cao et al. 2012). As a result, the down-regulation of oxytocin after sevoflurane exposure could imply a compromised protective mechanism that predisposes hippocampal neurons to noxious influences.

Social interaction in rodents is roughly achieved by olfactory investigation. Although social behaviors are varied, most of those behaviors involving social interaction are based on social recognition and discrimination abilities. These abilities are by and large subject to OT and AVP regulation in the hippocampus (Bielsky et al. 2004; Choleris et al. 2009; Dantzer et al. 1987; Ferguson et al. 2002; Winslow and Insel 2004).

Pharmacological studies in the past confirmed that AVP can enhance social recognition memory (Boccia et al. 1998; Caldwell et al. 2008). In AVP-receptor (AVPR) knockout mice, social memory deficits and abnormal emotional changes were observed (Bielsky et al. 2004). Brattleboro rats carrying a spontaneous null mutation of the AVP gene showed innate impairment in social recognition memory, similar to that of AVPR knockout mice. Moreover, AVP administration into the CNS restored their social memory (Engelmann and Landgraf 1994). OT, which is equally important as AVP, serves a similar function in the CNS to facilitate social recognition memory (Bielsky et al. 2004; Ferguson et al. 2001; Ross and Young 2009). In mice lacking the OT gene, social amnesia was observed (Ferguson et al. 2000; Lee et al. 2009). In humans, nasal administration of OT significantly improved the ability to understand people’s feelings from facial clues (Domes et al. 2007; Guastella et al. 2010), increased the duration of eye contact (Guastella et al. 2008a, b), and elevated the ratings of people’s facial trustworthiness and attractiveness (Di Simplicio et al. 2009; Guastella et al. 2008a, b; Theodoridou et al. 2009). Modahl et al. reported that plasma OT levels in children with social behavioral disorders were lower than those of age-matched controls (Modahl et al. 1998); these lower levels of OT might be due to the impaired processing of OT precursor (Green et al. 2001).

Recent studies have suggested social behavioral disorders may involve epigenetic modifications (Miyake et al. 2012; Schanen 2006), including methylation of certain genes (Jones et al. 2008). In 2009, Gregory et al. (2009) used genome-wide arrays to identify gene variants in 119 patients from families with a history of autism. A deletional mutation in the OT receptor (OTR) gene was discovered in one patient, who had one affected sibling with hypermethylation in the promoter of the OTR gene (Gregory et al. 2009). Hence, it can be inferred that hypermethylation of the OTR gene promoter leads to its low expression. To further examine this, researchers investigated DNA methylation at this region in 20 autistic patients and 20 controls. Their results revealed that patients with social behavior disorders had significantly higher methylation statuses at all three CpG sites of the OTR gene promoter (Gregory et al. 2009). Thus, it is now worth considering whether sevoflurane administration induces the methylation of the OT and AVP gene promoters to down-regulate their transcription.

The above evidence indicates that it is highly possible that the observed impairments of juvenile social recognition and discrimination abilities are closely related to the altered hippocampal OT and AVP expression after early sevoflurane exposure.

Studies in rodents have confirmed that during normal postnatal development, OT mRNA production may rise in the brain by many fold until puberty, with equal levels in both sexes; no major discrepancy was found between age-matched male and female mice during this period (Burbach et al. 1992; Chitme and Hiremath 2009). Protein concentrations and distributions of OT and OTR in the CNS are also of the same levels before puberty, as examined in both male and female rodents (Burbach et al. 1992; Lee et al. 2009; Pedersen et al. 1992). However, during estrus cycles, pregnancy, parturition, and lactation, considerable fluctuations of OT levels occur in female adults (Burbach et al. 1992; Gimpl and Fahrenholz 2001). Therefore, most behavioral studies in adult rodents only investigate male subjects.

A previous study revealed that in adult mice, neonatal exposure to sevoflurane impaired social memory (Satomoto et al. 2009). However, no study has ever investigated the effects of sevoflurane on the impairment on juvenile social behavioral abilities, especially on recognition and discrimination abilities, even though these basic properties are critical for the development of other social behaviors at later ages. However, from the research results so far, it is evident that sevoflurane down-regulates the activities of OT and AVP in the brain; these changes are accompanied by impaired juvenile social recognition and discrimination abilities.

## Abbreviations

OT: Oxytocin
AVP: Arginine vasopressin
IEI: Inter-encounter interval
PCR: Polymerase chain reaction
qPCR: Quantitative polymerase chain reaction
GAPDH: Glyceraldehyde phosphate dehydrogenase
RNA: Ribonucleic acid
mRNA: Messenger ribonucleic acid
PMSF: Phenylmethylsulfonyl fluoride
SDS: Sodium dodecyl sulfate
TEMED: Tetramethylethylenediamine
PB: Phosphate buffer
EDTA: Ethylenediamine tetraacetic acid
